# Assessment of oxidative stress parameters in HIV infection

**DOI:** 10.1186/1471-2334-14-S3-E28

**Published:** 2014-05-27

**Authors:** Shreewardhan Rajopadhye, A Rosalind Marita, MA Ansari, Abhay Chowdhary, Sucheta Dandekar

**Affiliations:** 1 Department of Biochemistry, Haffkine Institute, Mumbai-400012, Maharashtra, India; 2 Department of Biochemistry, Seth G.S.Medical College & KEM Hospital, Parel, Mumbai-400012, Maharashtra, India; 3 Department of Pathology, Seth G.S.Medical College & KEM Hospital, Parel, Mumbai-400012, Maharashtra, India

## Background

Both viral and host factors are responsible for oxidative stress in HIV disease, which in turn activates the replication of HIV provirus by various pathways. Oxidizing stress is a pathologic phenomenon resulting from imbalance between the system producing active oxygen species and those defending the organism. The present study was aimed to assess oxidative stress markers in HIV patients.

## Methods

The study included 30 HIV sero-positive patients, 30 healthy volunteers served as controls. Patients were categorized on the basis of their absolute CD4 counts into 3 groups - Group-1 (>500 CD4 cells/mm^3^), Group-2 (200–499 CD4 cells/mm^3^), and Group-3 (<200 CD4 cells/mm^3^).Lipid peroxidation was estimated using serum malondialdehyde as a marker, serum nitric oxide levels were assessed by Griess reagent method, serum reduced GSH by Beutler *et al*, serum C reactive protein, serum AOPP by Witko Savark method and serum proteins by Bradford method. Statistical analysis was done using the Student’s t test and one-way ANOVA.

## Results

Significant decrease (*p*<0.004) in GSH levels and significant increase (*p*<0.0008) in NO levels was observed in HIV infected group when compared to controls. However, no significant changes were found in levels of AOPP, MDA, and CRP in the study groups. Significant increase (*p*<0.0001) in MDA levels in group 3and in GSH levels (*p*<0.0395) in all 3 groups was seen as compared to controls.

## Conclusion

The findings indicate that considerable amount of oxidative stress are induced and changes in NO and GSH levels may contribute to the immunopathophysiology during HIV infection.

